# Lagged effects of childhood depressive symptoms on adult epigenetic aging

**DOI:** 10.1017/S0033291724001570

**Published:** 2024-09

**Authors:** Laura K. M. Han, Moji Aghajani, Brenda W. J. H. Penninx, William E. Copeland, Karolina A. Aberg, Edwin J. C. G. van den Oord

**Affiliations:** 1 Department of Psychiatry, Amsterdam UMC, location Vrije Universiteit, Amsterdam Neuroscience, Amsterdam, The Netherlands; 2 Centre for Youth Mental Health, The University of Melbourne, Parkville, VIC, Australia; 3 Orygen, Parkville, VIC, Australia; 4 Institute of Child & Education Studies, Section Forensic Family & Youth Care, Leiden University, The Netherlands; 5 Department of Psychiatry, University of Vermont, Burlington, USA; 6 The Center for Biomarker Research and Precision Medicine, School of Pharmacy, Virginia Commonwealth University, Richmond, VA, USA

**Keywords:** depressive symptoms, DNA methylation, epigenetic aging

## Abstract

**Background:**

Cross-sectional studies have identified health risks associated with epigenetic aging. However, it is unclear whether these risks make epigenetic clocks ‘tick faster’ (i.e. accelerate biological aging). The current study examines concurrent and lagged within-person changes of a variety of health risks associated with epigenetic aging.

**Methods:**

Individuals from the Great Smoky Mountains Study were followed from age 9 to 35 years. DNA methylation profiles were assessed from blood, at multiple timepoints (i.e. waves) for each individual. Health risks were psychiatric, lifestyle, and adversity factors. Concurrent (*N* = 539 individuals; 1029 assessments) and lagged (*N* = 380 individuals; 760 assessments) analyses were used to determine the link between health risks and epigenetic aging.

**Results:**

Concurrent models showed that BMI (*r* = 0.15, *P*_FDR_ < 0.01) was significantly correlated to epigenetic aging at the subject-level but not wave-level. Lagged models demonstrated that depressive symptoms (*b* = 1.67 months per symptom, *P*_FDR_ = 0.02) in adolescence accelerated epigenetic aging in adulthood, also when models were fully adjusted for BMI, smoking, and cannabis and alcohol use.

**Conclusions:**

Within-persons, changes in health risks were unaccompanied by concurrent changes in epigenetic aging, suggesting that it is unlikely for risks to immediately ‘accelerate’ epigenetic aging. However, time lagged analyses indicated that depressive symptoms in childhood/adolescence predicted epigenetic aging in adulthood. Together, findings suggest that age-related biological embedding of depressive symptoms is not instant but provides prognostic opportunities. Repeated measurements and longer follow-up times are needed to examine stable and dynamic contributions of childhood experiences to epigenetic aging across the lifespan.

## Introduction

During recent years, there has been a rapid increase in the number of studies examining ‘biological age’. Biological age is different from chronological age because it reflects the individual's biological state, rather than the time that has passed since birth. Promising and accurate indicators of biological age are based on DNA methylation levels. These ‘first-generation epigenetic clocks’ track the aging process with correlations between DNA methylation predicted age (*DNAm age*) and chronological age of typically over 0.90 (Horvath & Raj, 2018). More recently, ‘second-generation’ epigenetic clocks have also been developed (Levine et al., 2018; Lu et al., 2019). Instead of being optimized for chronological age prediction, these clocks focus on enhancing the prediction for aging- and mortality-related outcomes. In both cases , the predicted DNAm age can be contrasted to chronological age to study whether individuals are biologically younger or older than expected based on chronological age. From here on we refer to this difference as ‘*epigenetic aging*’.

Studies have shown associations between epigenetic aging *v.* age-related morbidities, and mortality (Chen et al., 2016). Correlations of epigenetic aging have also been found with several *health risks* such as traumatic stress (Wolf et al., 2018), depression (Han et al., 2018; Whalley et al., 2017), bipolar disorder (Fries *et al*. 2017), alcohol use disorder, metabolic syndrome components (Luo et al., 2020; Quach et al., 2017), body mass index (BMI), and socioeconomic factors (Schmitz et al., 2022; Simons et al., 2016). The broader literature thus suggests that psychiatric problems, lifestyle, and adversities are important health risks associated with epigenetic aging in adults (Gassen, Chrousos, Binder, & Zannas, 2016), as well as in children and adolescents (Colich, Rosen, Williams, & McLaughlin, 2020; Marini et al., 2020). Robust associations between various dimensions of socioeconomic status and several epigenetic clocks (e.g. GrimAge, DunedinPoAm, Levine, Yang clocks) have also been found in older adults (Schmitz et al., 2022). Similarly, environmental adversity, major depression, and functional impairments in developing adolescents were associated to a brain-based biological aging indicator (Drobinin et al., 2021), indicating the broad impact adversities may have on (multiple) biological systems. However, as most studies are cross sectional, it remains to be elucidated if health risks make epigenetic clocks ‘tick faster’ (i.e. accelerate biological aging).

Alternatively, the direction of effects may be reversed, or associations may be caused by ‘third’ variables that affect both the health risks and epigenetic aging. For example, individuals who are more prone to health problems may also be predisposed to age faster biologically due to genetic or environmental factors. Studying potential causal contributors to epigenetic aging is critical and may ultimately inform prevention and treatment regimens, especially for younger populations when age-related comorbid conditions have not manifested yet (Moffitt, Belsky, Danese, Poulton, & Caspi, 2017). Further longitudinal research is thus needed to disentangle the temporal links, including stable and dynamic contributions of adverse childhood experiences to biological aging indicators across the lifespan.

Longitudinal studies have the potential to shed light on the causal relationship between health risks and epigenetic aging, because they provide the unique opportunity to study within-person changes instead of between-person changes. Although it still does not prove causation, our longitudinal study can falsify causality and allows for stronger inferences by removing between-subject confounders. Existing studies have mainly focused on epigenetic aging trajectories over time (Li et al., 2020; Marioni et al., 2019) rather than studying the causal role of contributing factors identified by cross-sectional studies (Ryan, Wrigglesworth, Loong, Fransquet, & Woods, 2020). Only a small number of longitudinal studies exist that distinguish predictors of accelerated epigenetic aging over time from mere correlates that reflect ‘a snapshot of cellular age at one time point’ (Morrison et al., 2019). For example, one study found that advanced DNAm age (based on the Hannum but not Horvath algorithm) at a certain timepoint predicted higher metabolic syndrome severity two years later (Morrison et al., 2019).

The current study examines within-person changes of DNAm age across almost a two-decade period, from childhood to adulthood, and explores its relation to a broad but non-exhaustive selection of cross-sectionally identified health risks associated with epigenetic aging. A concurrent approach will be tested to examine whether within-person changes of health risks at a particular wave are accompanied by a within-person change in epigenetic aging at that same wave (i.e. wave-level correlation). Alternatively, it might also be possible that changes in health risks are only followed by changes in epigenetic aging later in life. For example, the initial impact of trauma may magnify over time due to subsequent increases in health-risk behaviors such as smoking, substance use, and high-risk activities (Bellis et al., 2019; Hughes et al., 2017) that then over time accelerate epigenetic aging. A lagged approach will therefore also be tested to examine whether exposure to unfavorable factors in childhood and adolescence predict a within-person change in epigenetic aging in adulthood.

## Methods

### The great smoky mountains study

The Great Smoky Mountain Study (GSMS) is a longitudinal study of 1420 participants from the southeast United States (Costello et al., 1996). GSMS started in 1993 when participants were children aged 9–13. Clinical data and blood spots were collected annually until age 16, and then again around ages 19, 21, 25, and 30. Although conducted separately, interviews were completed by both a parent figure and the participant until the age of 16. After 16 years, interviews were conducted with the participant only.

Sample selection for the current study was based upon availability of biological sample in both childhood (ages 9–16) and adulthood (ages 19–30) and having a range of exposure to childhood adversity levels. In total, *n* = 539 individuals were included in the concurrent analysis (*n* = 1039 assessments) and *n* = 380 individuals (*n* = 760 assessments) were included in the lagged analysis. The study was approved by Institutional Review Boards at Duke University and Virginia Commonwealth University and both parents and participants signed informed consent or assent forms.

### Measures

#### Body mass index

Data on height and weight were measured during interviews. BMI was assessed as kg/m^2^, where kg was a person's weight in kilograms and m^2^ their height in meters squared.

#### Psychiatric problems and trauma

Depressive and anxiety symptoms were assessed as follows: before age 16, both the child and parent completed a structured clinical interview using the Child and Adolescent Psychiatric Assessment (CAPA) (Angold and Jane Costello, 2000). After age 16 the Young Adult Psychiatric Assessment (YAPA) (Angold et al., 1999), the upward extension of the CAPA, was used. Depressive and anxiety symptoms were reported with a 3-month recency. Childhood trauma exposure was assessed by taking the sum of events concerning exposure to violence, sexual trauma, and other injury or trauma (online Supplementary Table S1). Impairments were assessed as a cumulative score of the total number of functional impairments 3 months prior to the interview, measured by summarizing dichotomous indicators across 17 areas of disrupted functioning, such as relationship with parents, teachers, peers, ability to complete chores at home, and disrupted schoolwork (Canino, Costello, & Angold, 1999).

#### Poverty

Being impoverished in childhood was parent-related and positive if the family met the poverty guidelines updated periodically in the Federal Register by the U.S. Department of Health and Human Services under the authority of 42 U.S.C. 9902(2). Being impoverished in adulthood was related to personal income. The poverty variable includes information from the Census Bureau's official poverty thresholds on income and inflation and is adjusted to standardize family size differences.

#### Substance use

Smoking tobacco, drinking alcohol, and using cannabis was also assessed. Either of the substance use categories were positive if reported through self- (adult) or parent (childhood) reports. Smoking was coded as one if the participant regularly smoked (i.e. at least one cigarette per day) in the past three months. Alcohol and cannabis were coded as one when daily/weekly use occurred or a use disorder for the substance was reported in the past 3 months.

#### Covariates

All health risks were available at all timepoints. Information on age (in years), biological sex assigned at birth and confirmed with omic data (male/female), Tanner pubertal stage (ordinal variable measured at each timepoint), and race/ethnicity was collected. Children completed a self-report measure of Tanner staging (Dorn, Susman, Nottelmann, Inoff-Germain, & Chrousos, 1990). Adult observations were coded as Tanner stage 5. More detailed information on the measures can be found in the *Supplement*.

#### Mean and rolling sums

Traditional imputation techniques do not make optimal use of longitudinal information or accommodate repeated measurements of time-dependent variables collected at irregular time intervals. To handle missing wave observations, we computed either a rolling sum or a mean across the waves the subject responded. Effectively, the use of a rolling sum is equivalent to assuming a score of zero for the missing waves and the use of the mean assumes the mean across all waves a subject responded or had a missing value. Thus, for health risks we used a rolling sum but for variables that cannot be zero (e.g. BMI) we used the mean.

### DNA methylation

Bloodspots were assayed for DNA methylation. Nearly all 28 million CpG sites in the methylome were assayed with an optimized protocol (Aberg et al., 2020; Han et al., 2018) for methyl-CG binding domain sequencing (MBD-seq). Elsewhere we summarized key features of the optimized MBD-seq approach using empirical data (Aberg, Chan, & van den Oord, 2020). We quality controlled reads, samples, and methylation sites. Data was processed and analyzed using the RaMWAS Bioconductor package (Shabalin, Clark, Hattab, Aberg, & van den Oord, 2017). The distribution of CD3^+^ (T-lymphocytes), CD14^+^ (monocytes), CD15^+^ (granulocytes), and CD19^+^ (B-lymphocytes) blood cells were estimated from the methylation data (Houseman et al., 2012) using reference methylomes specifically generated for this purpose (Hattab et al., 2017). For more details on the methylation assay, see *Supplement*. It is important to note that commonly used methods for assaying DNA methylation (and calculating DNA methylation age) often depend on Illumina arrays. These platforms generate variables indicating the percentage methylated (data ranges from 0 to 1). However, the current study used Methyl-CpG binding domain sequencing, generating methylation data that is semi quantitative (scores may range from 0 to 20). The currently used affinity-based capture methods have more comprehensive coverage of the methylome (interrogation of 94% of CpG sites), albeit at the cost of lacking single base resolution of bisulfite sequencing.

#### DNA methylation age

Following standard methods (Copeland, Shanahan, McGinnis, Aberg, & van den Oord, 2022; Han et al., 2018), we used elastic nets, with parameter alpha set to zero (i.e. ridge regression) (Friedman, Hastie, & Tibshirani, 2010), to predict chronological age (in years) from all methylome profiles. To obtain an unbiased estimate of the predictive power, k-fold cross-validation was used, with *k* = 10. Of the k subsets, *k*  − 1 were used as a ‘training set’ to fit the elastic net and obtain regression coefficients. The regression coefficients were then used to estimate chronological age for participants in the ‘test set’. By repeating this cycle of training and testing for each subset, age estimates are obtained for all participants. To avoid analyzing all methylation sites, of which the majority will not be associated with outcome and only add ‘noise’ to the model, we increased the number of sites included in the elastic net in steps until the predictive power did not increase anymore. The entire cycle of site selection through methylome-wide association studies followed by training the elastic net is repeated for each of the *k* folds. Because both the selection of sites and estimation of the prediction model is not affected by the participants in the test set, this yields an unbiased estimate of the predictive power. To evaluate predictive power we calculated: (a) the mean absolute error (MAE), (b) Pearson correlation coefficient between predicted DNAm age and chronological age, and (c) variance explained by the model (*R*^2^ ‘traditional formula’ function implemented in the *caret* package accounting for systematic over- and underestimations) (Kuhn, 2012).

### Statistical approach

#### Concurrent analyses of epigenetic aging

Since we have data of a group of individuals (i.e. *subjects*) measured at multiple time points (i.e. *waves*), several steps were taken to better understand the sources of covariance between epigenetic aging and the health risks. Intuitively speaking, we first calculated the *total covariance* between each health risk and epigenetic aging across all subjects and waves. For example, the total covariance between BMI and epigenetic aging represents how these two variables change together *across all subjects and waves*. Estimates were standardized by the total variance to obtain correlations. *Subject-level contribution* refers to the part of the relationship between each health risk and epigenetic aging that is due to *differences between individuals*. In other words, if individuals consistently have been exposed more to health risks than others, and this relates to differences in their epigenetic aging, this is considered the subject-level contribution. For example, if individuals who naturally have higher BMIs also tend to have higher epigenetic aging, this suggests a subject-level contribution. *Wave-level contribution*, on the other hand, refers to how each health risk and epigenetic aging changes together as individuals age over time. If, as individuals grow older, their epigenetic aging tends to increase or decrease in parallel to changes in health risks, that's the wave-level contribution. For example, if individuals generally experience an increase in BMI as they age, and this increase is associated with changes in their epigenetic aging, that suggests a wave-level contribution. The wave-level contribution complements the subject-level contribution to the total covariance between health risks and epigenetic aging. By decomposing the covariance between epigenetic aging and health risks into subject- and wave-level contributions, we gain insights into the underlying reasons for their relationship. If the subject-level contribution is significant, it suggests that inherent differences between individuals (e.g. genetics) play a substantial role in how epigenetic aging and health risks such as BMI are related. If the wave-level contribution is significant, it implies that changes in epigenetic aging and BMI are closely connected over time. *A significant wave-level contribution is necessary but not sufficient to infer that health risks have a causal effect on epigenetic aging.* Technically speaking, we used the *nlme R* package to fit bivariate mixed models (see Chapter 14 in (Rasbash, Browne, & Steele, 2003)). For modelling details, we refer to the *Supplement*.

#### Lagged changes in epigenetic aging

To assess whether exposure to a specific health risk in childhood/adolescence predicted a change in DNAm age between childhood and adulthood, we selected the latest available pre-adult (T2) and the adult (T3) DNA methylation assessment, excluding single or earlier pre-adult assessments (T1) for individuals with DNA methylation data from more than two timepoints available. The rationale for selecting the latest pre-adult time point is to capture the largest cumulative history of exposure to health risks before adulthood. We then fitted linear regression models with the change in *DNAm age* (DNAm age at T3 minus DNAm age at T2) as outcome and each health risk as predictor, resulting in ten separate models. To account for age effects, the adult age (i.e. chronological age at T3) and the change in chronological age (i.e. age at T3 minus age at T2) were included as covariates in the models, the resulting metric thus indicates *epigenetic aging*.

To examine whether epigenetic aging in childhood/adolescence predicted changes in health risks, we also fitted regression models with epigenetic aging at childhood/adolescence as predictor and change in health risk (e.g. BMI in adulthood minus BMI at the latest available childhood/adolescent observation) as an outcome, while correcting for health risk values at childhood/adolescence.

In all analyses, raw DNAm age was residualized for chronological age, while also accounting for the shared variance between age and other covariates in the model (Krieger et al., 2023). Continuous variables with values >3 × s.d. away from the mean were winsorized back to this threshold. All analyses (concurrent and lagged) included sex, race/ethnicity, estimated cell-type proportions, and lab technical covariates. For lagged analyses, we took the delta change in covariates between the adult and pre-adult time points to account for time-varying confounders, in addition to adding the covariates from the adult time point. Two-sided tests were performed and findings were false discovery rate (FDR) corrected using the Benjamini-Hochberg procedure (Benjamini & Hochberg, 1995) and considered statistically significant at *p* < 0.05. Model specifications and R code for analysis can be found on GitHub**.**

## Results

### Participant characteristics

Demographics and assessed phenotypes of the current study sample can be found in Table 1, while a breakdown per wave can be found in online Supplementary Table S2. Briefly, participants had one to three DNAm age assessments available, resulting in *n* = 1029 measurements from *n* = 539 participants (mean number of measurements per individual was 1.9) that were used for the concurrent analysis. Of these, *n* = 380 were included in the lagged analysis. Online Supplementary Figure S1 shows the chronological age distributions of individuals with one (*n* = 539, mean = 17.54 years, s.d. = 5.20, range = 9.47–31.66), two (*n* = 296, mean = 18.78 years, s.d. = 5.71, range = 9.07–33.31, mean follow-up time 9.84, s.d. = 4.00), or three measurements (*n* = 97, mean = 17.15 years, s.d. = 6.92, range = 9.01–34.55, mean follow-up time 1.98 years [s.d. = 1.69] between the first and second measurement, 12.22 years [s.d. = 4.77] between the second and the third, and 14.20 years [s.d. = 4.45], between the first and the third measurement). Online Supplementary Figure S2 displays pairwise correlations of the health risks across repeated measurements. In short, BMI showed the highest correlations over time (*r* between 0.61–0.78), followed by childhood trauma ((*r* between 0.20–0.68), and smoking ((*r* between 0.22–0.42). Relatively low correlations ((*r* < 0.28) were observed for the other health risks over time, suggesting that these health risks may reflect more acute rather than chronic exposures.

**Table 1.** Characteristics of samples included in concurrent and lagged analyses

		Concurrent analysis		Lagged analysis	
Characteristic	N*	mean ± s.d. (range)/n (%)	N*	Timing	mean ± s.d. (range)/n (%)
*Demographics*	539/1029		380/760		
Chronological age (years)		18.14 ± 6.05 (9.02–34.55)		Δ	10.74 ± 4.04 (2.70–20.46)
Predicted DNAm age (years)		18.16 ± 4.92 (8.00–33.19)		Δ	7.71 ± 3.26 (−0.99-17.36)
Sex (female)		521 (51%)		-	184 (48%)
*Race/ethnicity*	539/1029		380/760	-	
White		661 (64.2%)			244 (64.2%)
Black		49 (4.8%)			18 (4.7%)
American Indian		319 (31%)			118 (31.1%)
*Estimated cell counts*	539/1029		380/760		
CD03		0.27 ± 0.06 (0.10–0.51)		Δ	0.01 ± 0.06 (−0.22-0.24)
CD14		0.11 ± 0.02 (0.04–0.17)		Δ	−0.01 ± 0.02 (−0.09–0.06)
CD15		0.53 ± 0.07 (0.31–0.75)		Δ	0.02 ± 0.08 (−0.22–0.26)
CD19		0.08 ± 0.04 (0.00–0.21)		Δ	−0.03 ± 0.04 (−0.19–0.09)
Body Mass Index (kg/m^2^)	537/968	25.31 ± 6.95 (12.73–47.25)	380/760	T2	24.23 ± 5.14 (17.55–35.65)
*Psychiatric problems & Trauma*	539/1029		380/760		
Depressive symptoms		0.85 ± 1.11 (0.00–5.00)		T2	2.86 ± 2.51 (0.00–9.00)
Anxiety symptoms		0.95 ± 1.69 (0.00–7.00)		T2	3.99 ± 4.11 (0.00–14.00)
Childhood trauma		1.03 ± 1.07 (0.00–4.00)		T2	0.75 ± 0.60 (0.00–2.00)
Impairments		1.00 ± 2.22 (0.00–10.00)		T2	0.97 ± 1.06 (0.00–3.55)
*Social environment*			380/760		
Poverty (yes)	518/959	279 (29%)		T2	0.33 ± 0.28 (0.00–0.89)
*Substance use*	539/1029		380/760		
Smoking (yes)		361 (35%)		T2	0.33 ± 0.81 (0.00–4.00)
Cannabis (yes)		180 (17%)		T2	0.05 ± 0.25 (0.00–2.00)
Alcohol (yes)		188 (18%)		T2	0.04 ± 0.20 (0.00–2.00)

*N indicates the number of individuals/measurements. Abbreviations: DNAm, DNA methylation. Statistic presented: mean across measurements ± s.d. (minimum-maximum); n (%). For the concurrent analysis, we used all available data points from each individual. T1 and single measurements were excluded from the lagged analysis. T2 timing indicates the latest available observation (<17 years old) before adulthood. Δ indicates the difference between T3 and T2.

### Estimating DNA methylation age

Online Supplementary Figure S3 illustrates the explained variance of chronological age by the elastic net as a function of the number of methylation sites included as predictors. Chronological age could be predicted with a correlation of *r* = 0.93, R^2^ = 0.85, MAE of 1.85 years (MAE*_weighted_* = ((*MAE* (1.85 *years*))/(*age range of sample* (27 *years*))); MAE*_weighted_* = 0.07 years)(Cole, Franke, & Cherbuin, 2019), indicating high accuracy, particularly considering the restricted age range (online Supplementary Figure S4).

### Concurrent changes in epigenetic aging

Table 2 shows the proportion of subject-level (*column 1*) and wave-level variance (*column 2*). The proportion of subject-level variance (i.e. intra-class correlation) of epigenetic aging and each health risk indicates stability over time. The overall covariance between epigenetic aging and health risks (*column 3*) was decomposed into subject- (*column 4*) and wave-level contributions (*columns 5*) and is also presented in Table 2. We found significant subject-level contributions for BMI (*r* = 0.15, P_FDR_ < 0.01), meaning that higher BMI was positively correlated to higher concurrent epigenetic aging. No significant wave-level contributions were found.

**Table 2.** Decomposition of covariance between epigenetic aging and health risks into subject- and wave-level contributions

	Proportion subject-level variance*		Proportion wave-level variance		Overall correlation	Subject-level contribution to the correlation			Wave-level Contribution to the correlation		
	Epigenetic aging	Health risk	Epigenetic aging	Health risk	R	r	P	P_FDR_	r	P	P_FDR_
BMI	0.45	0.80	0.55	0.20	0.15	0.15	**<0.001**	**<0.01**	0.00	0.86	0.94
Depressive symptoms	0.44	0.24	0.56	0.76	−0.01	−0.01	0.65	0.65	0.00	0.77	0.94
Anxiety symptoms	0.44	0.23	0.56	0.77	0.06	0.04	0.22	0.39	0.02	0.09	0.32
Childhood trauma	0.44	0.50	0.56	0.50	0.08	0.06	0.07	0.32	0.02	0.11	0.32
Impairments	0.44	0.16	0.56	0.84	0.01	0.02	0.49	0.61	−0.01	0.35	0.53
Poverty	0.44	0.23	0.56	0.77	0.04	0.04	0.26	0.39	0.00	0.94	0.94
Smoking	0.44	0.32	0.56	0.68	0.05	0.04	0.17	0.38	0.01	0.27	0.49
Cannabis	0.44	0.09	0.56	0.91	0.07	0.04	0.15	0.38	0.03	0.04	0.32
Alcohol	0.44	0.02	0.56	0.98	0.04	0.02	0.54	0.61	0.02	0.14	0.32

BMI, Body Mass Index. All models were corrected for linear and quadratic age terms, sex, Tanner pubertal stage, race/ethnicity, estimated cell counts, and lab technical covariates. Significant *p*-values <0.05 are indicated in bold. P_FDR_ indicate false discovery rate adjusted *p*-values. *The proportion of subject-level variance is also known as the intra-class correlation and indicates stability over time.

### Lagged changes in epigenetic aging

**Figure 1.**
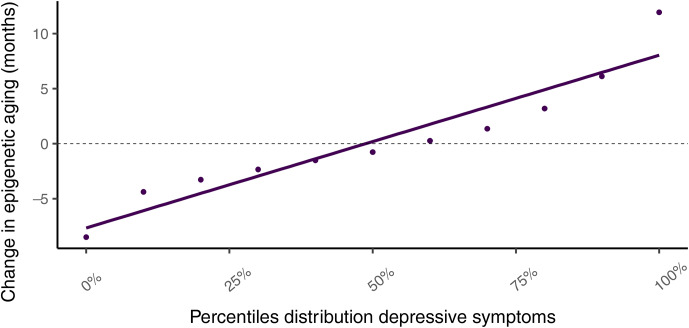
Fully adjusted lagged effects of depressive symptoms on epigenetic aging. The *x*-axis shows percentile distributions of the depressive symptoms, and the *y*-axis shows the change in epigenetic aging over time between two assessments in months. For example, persons with a depressive symptom score in the 99^th^ percentile showed 10 months of epigenetic aging on average. Models were residualized for Δ chronological age, adult age, sex, race/ethnicity, Δ body mass index, adult body mass index, Δ smoking, adult smoking, Δ cannabis, adult cannabis, Δ alcohol, adult alcohol, Δ estimated cell-type proportions, adult estimated cell-type proportions, and lab technical covariates.

For the lagged analysis, we used the latest observation before adulthood (9–16 years old) and the adulthood observation (18–35 years old), resulting in *n* = 380 individuals with two observations. The mean follow-up time for this analysis was 10.74 years (s.d. = 4.04). Results for the lagged effects of the health risks on epigenetic aging can be found in Table 3. Higher depressive symptoms (*b* = 1.67 months, *p* = 0.003) and functional impairments (*b* = 3.18 months, *p* = 0.012) were associated with higher rates of epigenetic aging (online Supplementary Figure S5). However, after multiple testing correction only depressive symptoms (P_FDR_ = 0.024) remained significant. To further explore the robustness of this significant finding, we post-hoc corrected this model for additional covariates pertaining to the within-person change in BMI, smoking, and cannabis and alcohol use. The fully corrected model showed a persistent significant lagged effect for depressive symptoms (*b* = 1.72 months, *p* = 0.005), Fig. 1. Persons with a depressive symptom score in the bottom 1st percentile were about 6 months younger than expected at their age, while those in the top 99th percentile showed 10 months of epigenetic aging on average. Online Supplementary Table S3 provides an overview of the epigenetic aging percentile distribution per health risk. There were no significant findings from the analyses examining whether epigenetic aging in childhood/adolescence predicted changes in health risks in adulthood (online Supplementary Table S4).

**Table 3.** Lagged effects of health risks on epigenetic aging

	b (in months)	95% CI	s.e.	t value	P	P_FDR_
BMI	−0.46	−0.99–0.07	0.27	−1.72	0.086	0.147
Depressive symptoms	1.67	0.59–2.76	0.55	3.03	**0.003**	**0.024**
Anxiety symptoms	0.56	−0.10–1.21	0.34	1.66	0.098	0.147
Childhood Trauma	4.43	−0.09–8.95	2.30	1.93	0.055	0.147
Impairments	3.18	0.71–5.65	1.26	2.53	**0.012**	0.053
Poverty	9.18	−0.97–19.33	5.16	1.78	0.076	0.147
Smoking	0.71	−2.60–4.02	1.68	0.42	0.673	0.757
Cannabis	7.90	−2.92–18.71	5.50	1.44	0.152	0.195
Alcohol	−1.83	−15.04–11.39	6.72	−0.27	0.786	0.786

b, unstandardized beta; 95% CI, 95% confidence intervals for regression coefficient b; SE, standard error of regression coefficient b; BMI, Body Mass Index. All models were corrected for Δ chronological age, adult age, sex, race/ethnicity, Δ estimated cell counts, adult estimated cell counts and lab technical covariates. Significant *p*-values <0.05 are indicated in bold. P_FDR_ indicate false discovery rate adjusted *p*-values.

## Discussion

In this study we used a unique longitudinal study design to gain further understanding of the concurrent and lagged effects of a wide range of health risks previously associated with more advanced epigenetic aging. The concurrent approach showed that BMI was correlated to epigenetic aging at the subject-level, consistent with previous literature (Gassen et al., 2016; Horvath & Raj, 2018). However, no evidence for wave-level contributions to the concurrent link between health risks and epigenetic aging was found, indicating that it is unlikely for health risks to instantly become biologically embedded. On the other hand, the lagged approach revealed that exposure to depressive symptoms predicted positive changes in epigenetic aging between childhood/adolescence and adulthood, while epigenetic aging in childhood/adolescence did not predict future health risks. Combined with the absence of wave-level contributions to the correlations in the concurrent models, we suggest that the effects of the studied health risks possibly require time to develop to be expressed later in life. Furthermore, this implies that improving mental health in children and adolescents may prevent accelerated or ameliorate accelerated biological aging in the future.

The relatively low correlations between health risk variables over time (online Supplementary Figure S2) suggest that most studied risk factors were dynamic. An alternative explanation for the lack of concurrent wave-level contributions is that wave-level correlations are confounded by measurement error, attenuating wave-level contributions to the covariance between health risks and epigenetic aging. It will be interesting to see whether a longer follow up time and older average age of our sample may result in significant concurrent effects. However, despite the dynamic variability of health risks, the detection of lagged effects indicates that initial exposure to depressive symptoms has a lasting impact on epigenetic aging. We argue that this trend may also be observed in the data for other considered health risks (i.e. anxiety symptoms, childhood trauma, functional impairments, and poverty) when considering a one-sided hypothesis in the direction of accelerated epigenetic aging (uncorrected for multiple testing), indicating the need to follow-up on these health risks in future studies with more statistical power. Such studies may also elucidate whether the significant lagged effects are *causal* and whether the other nonsignificant health risks may need more time to magnify and accelerate epigenetic aging. Together, these findings underscore the necessity of considering both the temporal dynamics of health risks and the importance of baseline measurements when studying the relationship between health risks and epigenetic aging.

Certain behavioral, psychiatric, and medical conditions frequently co-occur with stress exposures, and covary with changes in DNA methylation (and biological processes) central to biological aging (Han et al., 2019b). Consequently, these conditions may introduce confounding effects. As previously mentioned, subsequent increases in health-risk behaviors such as unhealthy lifestyle and substance use are commonly theorized to exacerbate the initial impact of stress exposures such as depression. The current study revealed robust predictive power of childhood depressive symptoms in pre-adulthood on accelerated epigenetic aging in adulthood that held its significance after additional adjustment for variables such as BMI, smoking, and the use of cannabis and alcohol. Nevertheless, it remains plausible that alternative underlying pathways currently not considered also exert influence. Perhaps those related to the physical environment as previously observed with, for example, water and air pollution (Alfano et al., 2018; Belsky & Baccarelli, 2023; Yannatos, Stites, Brown, & McMillan, 2023) or other shared genetic and environmental confounds at the family-level (Ingram et al., 2024). Overall, the present study raises the possibility that changes in depressive symptoms represent an early opportunity to examine change in future epigenetic aging and that epigenetic aging may potentially be a dynamic marker that is able to respond to environmental factors, if only as a proxy.

In contrast, our study did not provide evidence supporting that epigenetic aging assessed during childhood or adolescence holds predictive value for subsequent changes in adult health risks. Plausible explanations for this finding include the possibility that our sample may have been too young to accrue significant epigenetic aging, or that the dynamic epigenetic changes related to development and puberty may have obscured differences in epigenetic aging (Almstrup et al., 2016; deSteiguer et al., 2023; Han et al., 2019a). Another possibility could be that epigenetic aging measured during earlier life stages is more strongly confounded by pre- and perinatal exposures, potentially highlighting the importance of further exploring genetic and pre-environmental factors (Bozack et al., 2023; Simpkin et al., 2016). Such investigations help determine whether epigenetic aging is a consequence or contributing factor to specific (pediatric) phenotypes (Wang & Zhou, 2021). More longitudinal studies in pre-adult cohorts are therefore needed to examine the prognostic potential of epigenetic aging assessed in childhood and adolescence.

Childhood trauma and early life adversities have consistently been associated with biological aging in previous studies. These associations have been observed using different epigenetic clocks, including ‘first’ (e.g. Hannum, Horvath) (Wolf et al., 2018), but also ‘second generation’ epigenetic clocks like GrimAge (Hamlat, Prather, Horvath, Belsky, & Epel, 2021). Moreover, we reported similar findings using the current method in the same cohort (Copeland et al., 2022) and across different cohorts (Han et al., 2018). However, the present study found that childhood trauma only predicted accelerated epigenetic aging in adulthood with marginal significance (*p* = 0.055) before multiple comparison correction, suggesting that further research is necessary to confirm its robustness. Future studies may focus on examining the specific conditions under which childhood trauma and early life adversities affect epigenetic aging, such as the timing of exposure in birth cohorts that include more nuanced exploration of sensitive developmental windows (Marini et al., 2020), as well as the different types of adversities that may have varying predictive effects (Rampersaud et al., 2022). In addition, as second-generation epigenetic clocks enhance predictive capabilities compared to their first-generation counterparts (Levine, 2020), utilizing them in analyses may yield stronger associations than currently observed. Additional research in these areas will provide a more comprehensive understanding of the complex relationship between childhood trauma, early life adversities, and epigenetic clocks.

### Strengths and limitations

First, we observed relatively small overall correlations between the studied health risks and epigenetic aging (range −0.01 to 0.15). With limited variance to decompose, subject- and wave-level contributions to the correlation were also modest. Future longitudinal studies including persons with, for example, more chronic and severe depression and anxiety may provide larger effect sizes for the concurrent analyses. However, the statistical rigor of our study and the fully adjusted model bolsters confidence in the robust lagged accelerating effects of depressive symptoms on epigenetic aging. Second, the sequencing-based DNA methylation data considered in the current study is inherently different from those obtained from Illumina platforms, preventing us from applying established epigenetic clocks. Being limited to MBD-seq data, this study lacks external out-of-sample validation of the trained algorithm. However, the current DNAm predictions were unbiased and no data leakage between training and testing samples occurred. While outside the scope of the current study, we acknowledge that more work is needed to determine the generalizability of findings using other validated epigenetic clocks (e.g. GrimAge) based on other DNA methylation platforms and to establish external validity of the current algorithm in other samples. Finally, the current study did not include ages beyond middle adulthood (i.e. >35 years), thereby limiting the generalizability of findings to a broader lifespan perspective. Nevertheless, the current study offers valuable insights into the longitudinal relationship between health risks and epigenetic aging.

The current study can be expanded, and its results may be followed up in several ways. For example, it is possible that the time lag currently studied did not match the time needed for certain health risks to become epigenetically embedded. Ideally, to distinguish correlates from causes of epigenetic aging (Nelson, Promislow, & Masel, 2020), individuals need to be tracked over longer periods of time with more frequent sampling of DNA methylation and health measurements to estimate their covariance with more precision (Moffitt et al., 2017). Further studies in datasets with DNA methylation measurements at more time points may potentially reveal significant lagged effects of health risks that yielded non-significant findings in this study. Finally, a natural progression of this work is to investigate which (causal) factors or biological mechanisms might be driving the lagged effects (e.g. inflammatory responses in depression).

## Conclusion

Previous cross-sectional studies have established correlations between epigenetic aging and a diverse set of variables (Ryan et al., 2020), albeit with various algorithms (e.g. Hannum and Horvath). The current study provides a longitudinal within-person investigation of both concurrent and lagged changes of several health risks associated with epigenetic aging identified from the literature. Changes in health risks at a particular wave were unaccompanied by concurrent changes in epigenetic aging. As a result, the current study provides limited evidence for immediate ‘accelerated’ epigenetic aging. However, the time lagged approach reveals prognostic value of depressive symptoms on future epigenetic aging. This suggests that interventions targeting depressive symptoms in children and adolescents may potentially prevent accelerated biological aging later in life.

## Supporting information

Han et al. supplementary material 1 Han et al. supplementary material

Han et al. supplementary material 2 Han et al. supplementary material

Han et al. supplementary material 3 Han et al. supplementary material

## Data Availability

The Great Smoky Mountains Study (GSMS) cohort has been archived on the Inter-university Consortium for Political and Social Research (ICPSR) at the University of Michigan (Umich), https://www.icpsr.umich.edu/web/ICPSR/studies/37221. The phenotypic data can also be requested by submitting an analysis plan to the data owners (WE Copeland). As agreed with NIH and approved by the local IRB, the data was scheduled to be deposited in the NIH controlled access repository dbGAP. However, dbGAP is currently full and as soon as the new NIH controlled access repository comes online data will be deposited. Code used for analyses can be found on https://github.com/ejvandenoord/bivariate_mixed_model. Code to calculate DNAm age from MBD-seq methylation data can be found on https://www.bioconductor.org/packages/release/bioc/html/ramwas.html.

## Supplementary material

The supplementary material for this article can be found at https://doi.org/10.1017/S0033291724001570.
