# Supplementary material for: Lagged effects of childhood depressive symptoms on adult epigenetic aging
Source: Psychol Med. 2024 Oct 7;54(12):3398–406. doi: 10.1017/S0033291724001570 (PMC11496221; doi:10.1017/S0033291724001570)
Supplement: Han et al. supplementary material 1 — Han et al. supplementary material [file S0033291724001570sup001.docx]

**SUPPLEMENTARY MATERIALS**

**Lagged effects of childhood depressive symptoms on adult epigenetic aging**

The supplementary file contains more detailed information on (1) quality-controlled reads, samples, and DNA methylation sites, (2) the assessed measures, and (3) bivariate mixed model specifications. The supplementary file also contains supplementary figures and tables. Tables include (1) Operationalization of the childhood trauma variable, (2) Participant characteristics breakdown per wave assessment, (3) Epigenetic aging percentile distribution per health risk, (4) Non-significant lagged effects of epigenetic aging on changes in health risks, (5) The overlap of CpGs included in the current, Hannum, and Horvath DNA methylation age predictors, and (6) An overview of the 25,000 CpG sites included in our DNA methylation age predictor. Supplementary figures display (1) Chronological age distributions, (2) Pairwise correlations of the health risks across repeated measurements, (3) Stepwise selection of methylation sites to be included in the DNA methylation age prediction model, (4) Chronological age plotted against predicted DNA methylation age, and (5) Uncorrected lagged effects of health risks on epigenetic aging.

**SUPPLEMENTARY METHODS**

**Quality control of MBD-seq data in GSMS**

*DNA extraction and QC*

DNA extraction was extracted from dry blood spots using the QIAamp DNA Mini Kit (Qiagen) and the concentration of double stranded DNA was assessed with the Qubit 2.0 Fluorometer (Invitrogen). Depending on the size of (the part of) the blood spot available the amount of extracted DNA spanned a broad range from ~10 ng to nearly 1 ug that was used for downstream methylomic analysis.

## **MBD-seq**

We used components of the MethylMiner™ Kit (Invitrogen) to enrich for DNA fragments with methylated CpGs (mCpG) via affinity purification with the methyl-CG binding domain protein (MBD2). DNA was sonicated to 100-150 bp using a Covaris S2 ultra-sonicator. For each capture reaction, 15 μL (10 μL per μg DNA) of prepared MBD-beads (10 μg/uL beads; 350 ng/uL MBD-biotin protein) was incubated with 1.5 μg of fragmented DNA (200 μL final volume in 1x Bind-Wash Buffer) for 1 hours, at room temperature, on an orbital shaker at 650 rpm. Each capture reaction was washed three times with 1x Bind-Wash Buffer. The bound methylated fragments were recovered in three pooled elutions of 500 mM NaCl buffer (25% High Salt Elution Buffer, 75% Low Salt Elution Buffer v/v), and purified by ethanol precipitation. This optimal protocol was empirically determined, where we found that increased wash stringency, and elution with a low salt concentration improves the sensitivity/specificity of the assay for loci with modest numbers of CpG sites, giving better methylome-wide representation (Aberg et al., 2017; Aberg, Chan, & van den Oord, 2020; Chan et al., 2017).

The MBD enriched fractions were used to generate indexed libraries with the TruSeq Nano DNA HT Library Prep Kit (Illumina). Libraries were size-selected using SPRI beads to obtain a mean insert size of 150 bp. The 75-bp single-end libraries were then pooled and sequenced on the NextSeq 500 using High-Output v2 chemistry (Illumina).

## **Quality control of methylation data and CpG score calculation**

Reads were aligned (build hg19/GRCh37) with Bowtie2 (Langmead & Salzberg, 2012) using a seed-and-extend approach combined with local alignment while allowing for gaps. Specifically, we used a 20 bp seed with zero mismatches. Rather than considering the entire read, local alignment was used to improve sensitivity by finding the maximum similarity score between the reference sequence and a substring of the extension that may be “trimmed” at both ends. Gaps were allowed to account for small indels.

We performed thorough quality control of samples, reads, and CpGs (Aberg *et al.*, 2012) using the RaMWAS Bioconductor package (Shabalin, Clark, Hattab, Aberg, & van den Oord, 2017), which is specifically designed for large-scale methylation studies. The complete set of methylation data included methylation profiles from 1,202 dry blood spots from 571 unique individuals. Of the generated methylation profiles, 10 were excluded due to failed libraries or sequencing (mainly poor library quality or low number of reads) and 41 profiles were excluded because of poor or failed enrichment (peak skewness, unexpected peak size and/or high background levels). After calling SNPs from the methylation sequencing reads using GATK (McKenna *et al.*, 2010), we tested for agreement of genotypes between different blood samples from the same subject (Costello *et al.*, 2013). We found 6 samples for which the genotype information did not match with the GWAS genotype information. This indicated that a sample swap, or sample contamination may have occurred. As it was impossible to determine whether the problem was caused by the GWAS or MWAS data, we conservatively excluded all six samples from further analysis. We used the R function ‘pcout’ in the ‘mvoutliers’ package (with the upper boundary for outlier detection set to 15, the scaling constant set to 0.5, and the boundary for final outliers set to 0.2) to identify multidimensional outliers using principal components of the methylation data as input. Fourteen samples were multidimensional outliers and omitted. Next, we compared methylation age, as estimated from the profiles (Han *et al.*, 2018), with reported chronological age. One sample swap was corrected. That is, for an individual the methylation age estimate indicated that the oldest and the youngest time points had been exchanged. The middle time point remained correct. Lab records further supported the correction of this swap. Furthermore, the reported sex in the phenotype files were checked for agreement with the overall amount of methylation detected on the sex chromosomes. No further sample swaps, or errors in the phenotype file were detected. However, for two samples, from a single individual, we observed a possible karyotypic abnormality of the sex chromosomes. Both samples from this individual were excluded from further analysis. Cell type proportion were estimated using a MBD specific reference panel (Hattab *et al.*, 2017) after cell sorting to obtain DNA from the four common cell-types in blood (Hattab *et al.*, 2017) : T-cells (CD3+), monocytes (CD14+), granulocytes (CD15+), and B-cells (CD19+). Houseman method (Houseman *et al.*, 2012) was used to generate priors (the estimated means and twice the estimated standard deviations) to obtain final estimates by empirical Bayes using the R rstanarm package. We used the estimated cell-type proportions and cell-type means in the reference panel to predict methylation levels in whole blood. These predicted methylation levels were correlated. A total of 84 samples were excluded because of low correlations (r<0.32). This left 1,045 samples, of which 11 samples did not have any phenotypic data, resulting in 1,034 samples. For this project we further limited the selection to individuals who had a maximum of 3 DNA methylation assessments, as we could not run random permutations while preserving the subject-level dependence due to lack of other participants with >3 observations. This left a total of 1,029 samples from 539 participants.

The mean number of reads for samples used in this study was 59.7 million (SD=7.4 million) of which, on average, 99% aligned. Aligned reads were checked for excessive duplicate reads (>3 reads starting at the same location were reset to 1) and reads located in loci where alignment is challenging, determined by an in-silico experiment described elsewhere (Aberg *et al.*, 2012), were excluded. This left an average of 49.4 million (SD=7.4 million) reads per sample (=82.7% of all reads).

To identify CpGs, we combined reference genome sequence (hg19/GRCh37) with common SNPs calculated on the European super population from 1000 Genomes (Phase 3). To avoid including sites that are CpGs in only a very small proportion of subjects, we excluded CpGs created by SNPs with minor allele frequency <1%. This resulted in 27,916,990 CpGs. CpGs in loci prone to alignment errors, e.g. in repetitive regions, were eliminated prior to the analysis. To identify these CpGs, we used RaMWAS to perform the in-silico alignment experiment outlined elsewhere (Aberg *et al.*, 2012). In this experiment, the vast majority of CpGs (89.3%) were located in regions that showed perfect alignment coverage and only 1.3% (365,223 CpGs) showed evidence of alignment problems defined as 15% or more reads from this locus not aligning properly. Finally, akin to filtering SNPs with low minor allele frequency, we excluded rarely methylated sites (average read coverage <0.3). This left 22,670,747 autosomal CpGs for MWAS, which corresponds to 81% of all common CpGs in the human genome.

## **Quantifying methylation**

A natural way to quantify methylation for MBD-seq is to count the number of fragments covering a CpG site. However, with single-end libraries the fragment sizes are not observed. Counting the number of reads instead, seriously underestimates the amount of methylation as the sequenced fragment is usually longer than the read. RaMWAS therefore first uses a non-parametric approach to estimate the fragment size distribution from the sequencing data using isolated CpGs (van den Oord *et al.*, 2013). The fragment size distribution is used to calculate the probability that a sequenced fragment will cover the CpG under consideration. For example, this probability is 1.0 for fragments with reads starting within one read-length of the CpG, but is ≤1.0 for fragments with reads starting more than one read-length away. The CpG score is then calculated by taking the sum of probabilities for all fragments aligning within proximity of the CpG.

**Cell type measurements**

To estimate cell type proportions, we used reference methylomes (Houseman *et al.*, 2012; Koestler *et al.*, 2013). Whole blood samples of six subjects were used to isolated cells with 5 clusters of differentiation (CD3, CD19, CD20, CD14, and CD15) that capture the most common cell types in blood (T-cells, B-cells, monocytes, and granulocytes). Cell populations were isolated by positive selection using EasySep™ kits (Stemcell technologies) that apply magnetic nanoparticles coated with antibodies against a particular surface antigen (CD molecules). All reference methylomes were generated using MBD-seq. In a previous paper we showed that the estimated cell type proportions effectively controlled for cell type heterogeneity in methylome-wide association studies (Hattab *et al.*, 2017).

**Assessed measures**

*Tanner Pubertal Status*

Self-ratings of pubertal status were made using Tanner stage pictorial assessments of breast and pubic hair development (Tanner, 1962). Such ratings show moderate correlations with physical examination based on Tanner stages (Dorn & Biro, 2011). With parental agreement, each child was provided with sex-appropriate schematic drawings and asked to rate their current status on each dimension. There was no evidence of differential associations of breast development and pubic hair with other pubertal measures (e.g., timing, sex steroid levels) in females, or external genitalia development and pubic hair in males. The mean of the two ratings (breast/external genitalia development and pubic hair) was used as an overall index of morphological development (ranging from I-V for pre-pubertal to full maturity).

*Cumulative childhood trauma exposure*

Childhood trauma was scored as present at a wave when either the parent or the child reported any event listed in **Supplementary Table S1.** Traumatic events include both events directly experienced by the participant but also those in which the participant was a witness. Non-violent and violent sexual abuse were both considered sexual abuse. To capture the full range of exposure and maximize statistical power (Cecil, Zhang, & Nolte, 2020), we summed the number of waves up to the childhood methylation assessments when childhood trauma was present. Childhood trauma was scored as present at a wave when either the parent or the child reported any event. Childhood was defined as age < 21. In the majority of waves the parent/child was queried about the occurrence of each event since the last interview. However, until wave 4 participants were asked about the lifetime occurrence of each event. To avoid counting the same event multiple times, the number of events for waves 1 to 4 could never exceed 1.

### **Supplementary Table S1.** List of events used to calculate the childhood trauma score

| Violence |
| --- |
| Violent Death of loved one |
| Violent Death of sib/peer |
| War, terrorism |
| Cause of death or severe harm |
| Victim of physical violence |
| Physical abuse by relative |
| Captivity |
| Sexual trauma |
| Sexual abuse |
| Rape |
| Coercion |
| Other Injury or Trauma |
| Diagnosis of Physical illness |
| Serious accident |

*Number of functional impairments*

Psychosocial impairment secondary to psychiatric symptomatology was also rated according to a series of criteria specified in the CAPA glossary and the interview schedule (Adrian Angold & Jane Costello, 2000). Broadly, some decrement in actual function had to be described for a positive rating to be given (see (A. Angold et al., 1995) for a full description of definitions and concept of impairment implemented in the CAPA). A cumulative score of the total number of functional impairments was measured by summarizing dichotomous indicators across 17 areas of disrupted functioning in areas such as relationship with parents, teachers, peers, ability to complete chores at home, and disrupted schoolwork (Canino, Costello, & Angold, 1999). Impairments have previously been related to emotional and behavioral reported symptoms.

# **Bivariate mixed model**

We have longitudinal data for DNAm age and the health risks of interest. This translates to a 3-level mixed model as responses Y are nested in wave (i.e., we have DNAm age and health risk information for each wave), which in turn is nested in subject (multiple longitudinal measurements per individual). Let $Y_{\mathrm{ijk}}$ be the response for variable *i*=1..2 (i.e., DNAm age and health risk), at wave *j* (*j*=1..for number of subjects) and subject *k* (*k*=1..number of subjects). Assume dummy variable D_1jk_ is coded 1 if for DNAm age and 0 for the health risk variable and dummy variable D_2jk_ = 1- D_1jk_ which is coded 1 if for the health risk variable and 0 for DNAm age. The model can now be written as:

$Y_{\mathrm{ijk}}=b_{\mathrm{ijk}}{D_{\mathrm{ijk}}+c_{i1}Age}+\sum_{p=2}^{n_{p}} c_{\mathrm{ip}}{Cov}_{p}$, with $b_{\mathrm{ijk}}=b_{i}+u_{\mathrm{ijk}}+v_{\mathrm{ik}}$

Chronological age (Age) needs to be regressed out so that we study the residuals of $Y_{1jk}$ that represent the biogical age of subject *k* at wave *j*. In addition, the model includes a set of $p=2..n_{p}$ other covariates (${Cov}_{p})$with fixed effects $c_{\mathrm{ip}}$ such as Age^2^, sex or lab technical variables.

To decompose the correlation between epigenetic aging and health risk factors in a subject and wave contribution, we first write the model as:

$Y_{1jk}=b_{1jk}{D_{1jk}+c_{11}Age}+\sum_{p=2}^{n_{p}} c_{1p}{Cov}_{p}$, with $b_{1jk}=b_{1}+u_{1jk}+v_{1k}$

$Y_{2jk}=b_{2jk}{D_{2jk}+c_{21}Age}+\sum_{p=2}^{n_{p}} c_{2p}{Cov}_{p}$, with $b_{2jk}=b_{2}+u_{2jk}+v_{2k}$

Assume,

$\left[ \begin{matrix} v_{1k} \\ v_{2k} \end{matrix} \right]\sim(N,\Omega_{v})$,$\Omega_{v}=\left[ \begin{matrix} \sigma_{v1} & \\ \sigma_{v12} & \sigma_{v2} \end{matrix} \right]$

Thus, $\sigma_{v1}$ represents the subject level contribution to the variance of DNAm age that remains after regressing out the covariates, $\sigma_{v2}$ represents the contribution to the variance of the health risk variable that remains after regressing out the covariates, and $\sigma_{v12}$ represents the subject level contribution to the covariance between DNAm age and the health risk variable after regressing out the covariates. Denote the total variance-covariance matrix after regressing out the $n_{p}$ covariates as:

$$\Omega_{t}=\left[ \begin{matrix} \sigma_{t1} & \\ \sigma_{t12} & \sigma_{t2} \end{matrix} \right]$$

We can now estimate the wave contribution to this overall variance-covariance matrix by subtraction:

$$\Omega_{t}-\Omega_{v}=\Omega_{u}=\left[ \begin{matrix} \sigma_{u1} & \\ \sigma_{u12} & \sigma_{u2} \end{matrix} \right]$$

where and $\sigma_{u12}$ represents the wave level contribution to the covariance between DNAm age and the health risk variable after regressing out the covariates. The estimates $\sigma_{v12}$and $\sigma_{u12}$ can be further standardized (e.g., $r_{v12}={\sigma_{v12}/\sqrt{\sigma_{v1}\sigma_{v1}}}$) to obtain subject- and wave-level contributions to the correlation between epigenetic aging and the health risk variable.

The model was estimated using the R nlme package by maximum likelihood, leaving the covariance structure unrestricted through a Cholesky decomposition. The outcome variables were randomly permuted to perform significance tests of $\sigma_{v12}$ and $\sigma_{u12}$

**SUPPLEMENTARY RESULTS**

**Participant Characteristics**

Chronological age distributions of the current sample are shown in **Supplementary Figure S1**. Participants either had between 1 and 3 methylation and linked health risk assessments collected from 18 different waves. Demographics and assessed health risks of the current study sample breakdown per wave assessment can be found in **Supplementary Table S2. Supplementary Figure S2** displays pairwise correlations of the health risks across repeated measurements. In short, BMI showed the highest correlations over time (𝑟 between 0.61-0.78), followed by childhood trauma (𝑟 between 0.20-0.68), and smoking (𝑟 between 0.22-0.42). Relatively low correlations (𝑟<0.28) were observed for the other health risks over time, suggesting that these health risks may have been more acute rather than chronic.


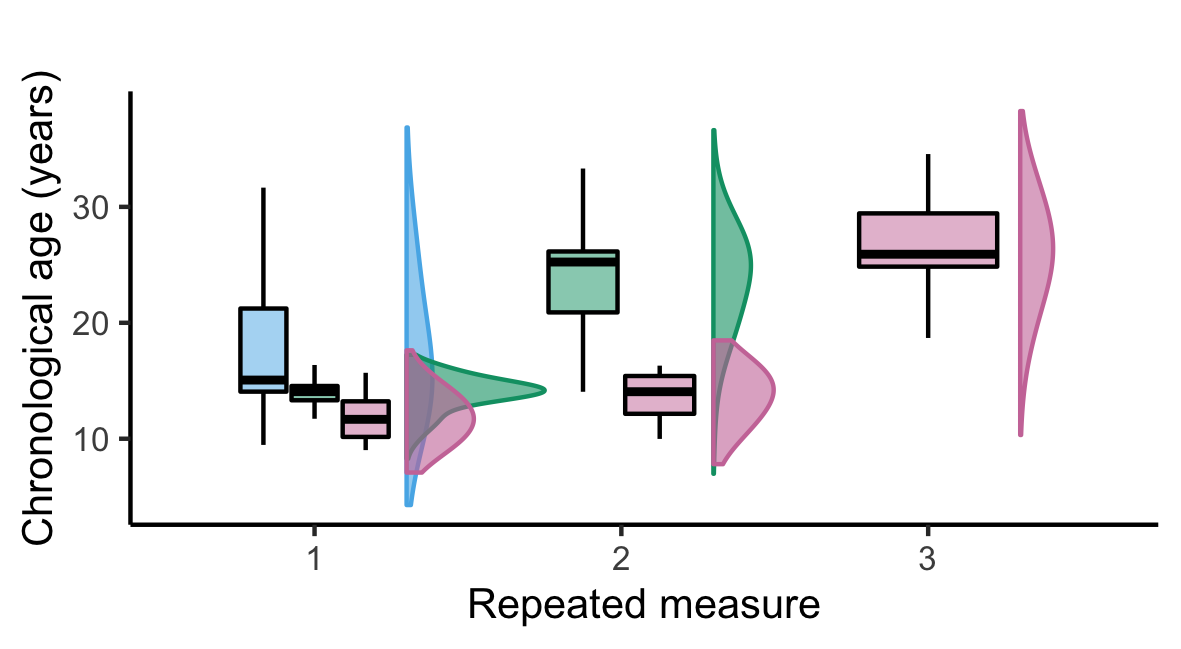


**Supplementary Figure S1. Chronological age distributions.** The plot shows individuals with one (blue, n=539), two (green, n=296), or three (pink, n=97) concurrent epigenetic aging and health risk measurements.

**Supplementary Table S2. Participant Characteristics and Number of Measurements Breakdown per Wave Assessment**

| **Characteristic** | **N** | **Wave 1**, N = 80 | **Wave 2**, N = 163 | **Wave 3**, N = 70 |
| --- | --- | --- | --- | --- |
| Chronological age (years) | 1,029 | 11.48 ± 1.66 (9.02-13.86) | 13.01 ± 1.64 (9.98-14.98) | 12.95 ± 1.59 (10.97-15.58) |
| Sex (Female) | 1,029 | 38 (48%) | 78 (48%) | 36 (51%) |
| Body Mass Index (kg/m^2^) | 968 | 21.19 ± 4.79 (13.03-33.17) | 22.84 ± 5.87 (14.43-41.47) | 21.12 ± 4.97 (12.73-36.98) |
| Depressive symptoms | 1,029 | 0.44 ± 0.69 (0.00-3.00) | 0.75 ± 0.93 (0.00-4.00) | 0.83 ± 1.26 (0.00-5.00) |
| Anxiety symptoms | 1,029 | 0.92 ± 1.45 (0.00-6.00) | 1.07 ± 1.74 (0.00-7.00) | 1.10 ± 1.73 (0.00-7.00) |
| Childhood trauma | 1,029 | 0.21 ± 0.41 (0.00-1.00) | 0.47 ± 0.50 (0.00-1.00) | 0.69 ± 0.47 (0.00-1.00) |
| Impairments | 1,029 | 0.70 ± 1.87 (0.00-10.00) | 1.06 ± 2.07 (0.00-10.00) | 1.91 ± 3.01 (0.00-10.00) |
| Poverty (yes) | 959 | 22 (29%) | 43 (28%) | 18 (29%) |
| Smoking (yes) | 1,029 | 3 (3.8%) | 21 (13%) | 14 (20%) |
| Cannabis (yes) | 1,029 | 1 (1.2%) | 3 (1.8%) | 2 (2.9%) |
| Alcohol (yes) | 1,029 | 0 (0%) | 3 (1.8%) | 3 (4.3%) |
| Statistics presented: mean ± SD (minimum-maximum); n (%); N reflects number of measurements. | | | | |

*(continued on next page)*

| **Characteristic** |  | **Wave 4**, N = 157 | **Wave 5**, N = 29 | **Wave 6**, N = 32 |
| --- | --- | --- | --- | --- |
| Chronological age (years) |  | 13.91 ± 1.21 (11.81-16.69) | 15.23 ± 0.32 (14.91-16.02) | 14.99 ± 1.03 (13.92-16.43) |
| Sex (Female) |  | 80 (51%) | 14 (48%) | 17 (53%) |
| Body Mass Index (kg/m^2^) |  | 23.94 ± 6.39 (14.20-47.25) | 22.84 ± 5.95 (16.16-45.60) | 26.42 ± 6.35 (17.02-42.29) |
| Depressive symptoms |  | 0.92 ± 1.02 (0.00-5.00) | 0.76 ± 1.06 (0.00-4.00) | 0.72 ± 0.89 (0.00-3.00) |
| Anxiety symptoms |  | 0.90 ± 1.60 (0.00-7.00) | 0.62 ± 1.01 (0.00-4.00) | 0.75 ± 1.61 (0.00-7.00) |
| Childhood trauma |  | 0.68 ± 0.47 (0.00-1.00) | 0.83 ± 0.80 (0.00-2.00) | 1.19 ± 0.86 (0.00-3.00) |
| Impairments |  | 1.69 ± 2.77 (0.00-10.00) | 1.83 ± 2.90 (0.00-10.00) | 1.69 ± 2.76 (0.00-10.00) |
| Poverty (yes) |  | 45 (31%) | 7 (25%) | 3 (11%) |
| Smoking (yes) |  | 25 (16%) | 7 (24%) | 7 (22%) |
| Cannabis (yes) |  | 2 (1.3%) | 3 (10%) | 3 (9.4%) |
| Alcohol (yes) |  | 0 (0%) | 1 (3.4%) | 1 (3.1%) |
| Statistics presented: mean ± SD (minimum-maximum); n (%); N reflects number of measurements. | | | |  |

*(continued on next page)*

| **Characteristic** |  | **Wave 7**, N = 65 | **Wave 8**, N = 16 | **Wave 9**, N = 59 |
| --- | --- | --- | --- | --- |
| Chronological age (years) |  | 16.18 ± 1.73 (14.92-19.95) | 16.17 ± 0.17 (15.94-16.44) | 20.12 ± 1.14 (18.78-21.77) |
| Sex (Female) |  | 32 (49%) | 9 (56%) | 24 (41%) |
| Body Mass Index (kg/m^2^) |  | 24.97 ± 5.75 (16.46-43.33) | 28.42 ± 7.97 (19.83-47.25) | 27.09 ± 6.96 (16.26-47.25) |
| Depressive symptoms |  | 1.14 ± 1.37 (0.00-5.00) | 1.56 ± 1.75 (0.00-5.00) | 0.90 ± 1.08 (0.00-5.00) |
| Anxiety symptoms |  | 1.17 ± 1.92 (0.00-7.00) | 1.81 ± 2.69 (0.00-7.00) | 0.88 ± 1.70 (0.00-7.00) |
| Childhood trauma |  | 0.83 ± 0.74 (0.00-2.00) | 1.69 ± 1.01 (0.00-4.00) | 0.97 ± 0.91 (0.00-4.00) |
| Impairments |  | 1.82 ± 2.77 (0.00-10.00) | 2.88 ± 3.54 (0.00-10.00) | 0.63 ± 1.41 (0.00-7.00) |
| Poverty (yes) |  | 24 (44%) | 2 (22%) | 33 (57%) |
| Smoking (yes) |  | 21 (32%) | 8 (50%) | 38 (64%) |
| Cannabis (yes) |  | 13 (20%) | 4 (25%) | 17 (29%) |
| Alcohol (yes) |  | 11 (17%) | 4 (25%) | 24 (41%) |
| Statistics presented: mean ± SD (minimum-maximum); n (%); N reflects number of measurements. | | | | |

*(continued on next page)*

| **Characteristic** |  | **Wave 11**, N = 44 | **Wave 13**, N = 26 | **Wave 14**, N = 70 |
| --- | --- | --- | --- | --- |
| Chronological age (years) |  | 20.09 ± 1.19 (18.31-21.95) | 21.08 ± 0.23 (20.73-21.49) | 26.44 ± 0.46 (25.98-28.39) |
| Sex (Female) |  | 23 (52%) | 11 (42%) | 42 (60%) |
| Body Mass Index (kg/m^2^) |  | 28.33 ± 7.68 (17.89-47.25) | 30.05 ± 7.70 (19.94-47.25) | 28.86 ± 6.57 (17.99-47.25) |
| Depressive symptoms |  | 0.98 ± 1.45 (0.00-5.00) | 0.81 ± 1.10 (0.00-5.00) | 0.93 ± 1.24 (0.00-5.00) |
| Anxiety symptoms |  | 1.00 ± 2.03 (0.00-7.00) | 0.73 ± 1.71 (0.00-7.00) | 0.89 ± 1.77 (0.00-7.00) |
| Childhood trauma |  | 1.41 ± 1.35 (0.00-4.00) | 1.92 ± 1.57 (0.00-4.00) | 1.41 ± 1.16 (0.00-4.00) |
| Impairments |  | 0.45 ± 1.69 (0.00-10.00) | 1.15 ± 2.85 (0.00-10.00) | 0.10 ± 0.64 (0.00-5.00) |
| Poverty (yes) |  | 17 (44%) | 12 (46%) | 9 (13%) |
| Smoking (yes) |  | 22 (50%) | 12 (46%) | 51 (73%) |
| Cannabis (yes) |  | 14 (32%) | 6 (23%) | 26 (37%) |
| Alcohol (yes) |  | 12 (27%) | 5 (19%) | 20 (29%) |
| Statistics presented: mean ± SD (minimum-maximum); n (%); N reflects number of measurements. | | | |  |

*(continued on next page)*

| **Characteristic** |  | **Wave 15**, N = 82 | **Wave 16**, N = 75 | | **Wave 17**, N = 22 | | **Wave 18**, N = 39 |
| --- | --- | --- | --- | --- | --- | --- | --- |
| Chronological age (years) |  | 25.57 ± 0.47 (23.51-26.73) | 25.41 ± 0.57 (24.45-26.47) | | 28.48 ± 1.48 (26.03-31.77) | | 30.96 ± 1.35 (29.37-34.55) |
| Sex (Female) |  | 44 (54%) | 45 (60%) | | 10 (45%) | | 18 (46%) |
| Body Mass Index (kg/m^2^) |  | 29.02 ± 7.32 (17.04-47.25) | 29.60 ± 7.02 (18.55-47.25) | | 25.60 ± 4.26 (16.33-34.48) | | NA |
| Depressive symptoms |  | 0.96 ± 1.20 (0.00-5.00) | 0.89 ± 0.92 (0.00-3.00) | | 0.55 ± 1.10 (0.00-5.00) | | 0.90 ± 1.35 (0.00-5.00) |
| Anxiety symptoms |  | 0.84 ± 1.58 (0.00-7.00) | 0.80 ± 1.19 (0.00-5.00) | | 0.32 ± 1.29 (0.00-6.00) | | 1.23 ± 2.30 (0.00-7.00) |
| Childhood trauma |  | 2.05 ± 1.36 (0.00-4.00) | 1.59 ± 1.42 (0.00-4.00) | | 1.59 ± 1.33 (0.00-4.00) | | 1.97 ± 1.06 (0.00-4.00) |
| Impairments |  | 0.30 ± 1.12 (0.00-7.00) | 0.08 ± 0.51 (0.00-4.00) | | 0.00 ± 0.00 (0.00-0.00) | | 0.18 ± 0.85 (0.00-5.00) |
| Poverty (yes) |  | 18 (22%) | 15 (20%) | | 6 (27%) | | 5 (15%) |
| Smoking (yes) |  | 52 (63%) | 47 (63%) | | 12 (55%) | | 21 (54%) |
| Cannabis (yes) |  | 37 (45%) | 27 (36%) | | 9 (41%) | | 13 (33%) |
| Alcohol (yes) |  | 36 (44%) | 30 (40%) | | 12 (55%) | | 26 (67%) |
| Statistics presented: mean ± SD (minimum-maximum); n (%); N reflects number of measurements. | | | |  | |  | |

**
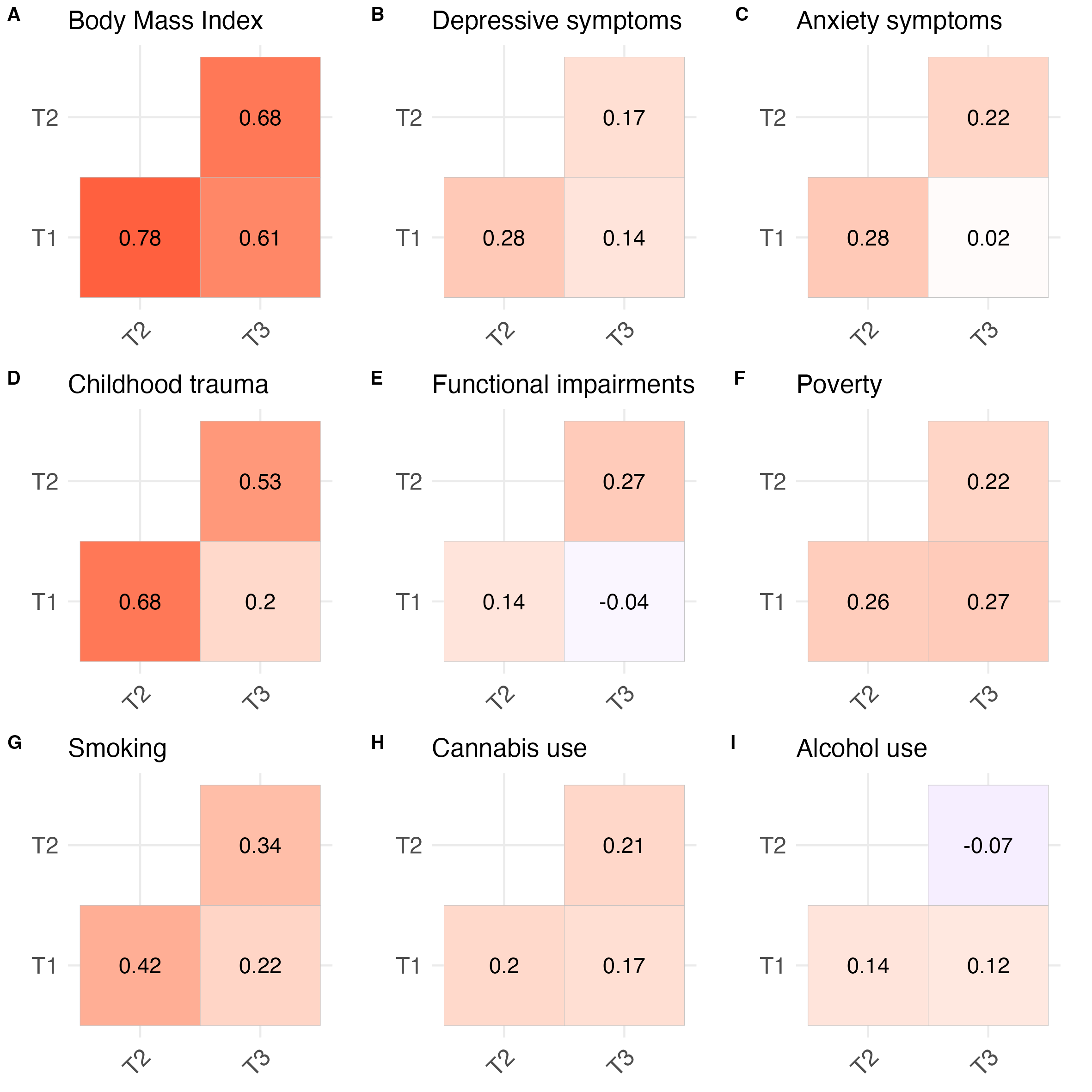
Supplementary Figure S2.** Pairwise correlations of the health risks across repeated measurements.

**DNA methylation age estimation**

To avoid analyzing all CpGs, of which the majority will not be associated with outcome and only add “noise” to the model, we increased the number of sites included in the elastic net in steps (100, 1000, 2500, 5000, 10 000, 25 000, 50 000, 75 000) until the explained variance of age did not improve anymore (**Supplementary Figure S3)**. We previously performed tests where the number of CpGs/genes was included in the loop over the k-folds. However, as it produced very similar results but is much more computer intensive (Clark *et al.*, 2019), this latter approach was not used. The correlation between predicted DNAm age and chronological age (r=0.93, R^2^=0.85, MAE=1.85 years) is depicted in **Supplementary Figure S4**.

**Supplementary Figure S3. Stepwise selection of methylation sites to be included in DNA methylation age estimator.** We increased the number of sites included in the elastic net in steps until the explained variance of chronological age did not improve anymore, resulting in 25,000 methylation sites included in the final prediction model (r=0.93 between chronological age vs. predicted DNA methylation age, R^2^=0.85).


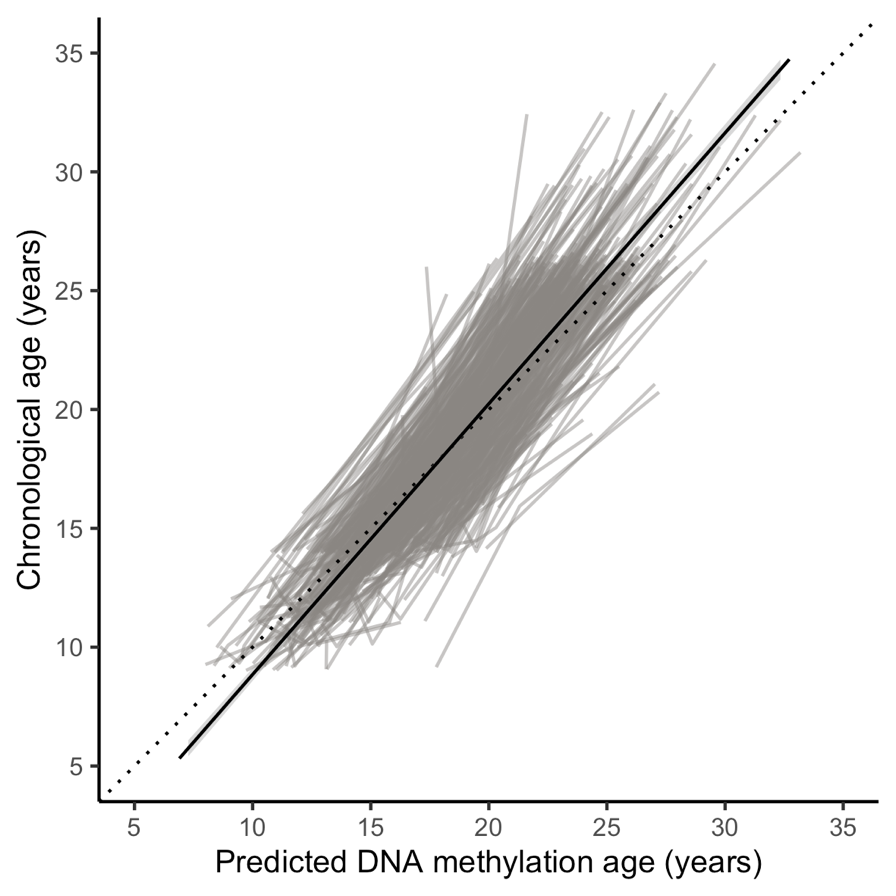


**Supplementary Figure S4. DNA methylation age.** Correlation between predicted DNA methylation age and chronological age (r=0.93, P<0.0001). Mean absolute error was 1.85 years. Diagonal dashed line reflects the line of identity (x=y). Each grey line represents one individual.

**Stability of epigenetic aging and health risks over time**

The proportion of subject-level variance (also known as the intra-class correlation) of epigenetic aging and each health risk indicates stability over time. BMI showed a high level of stability (i.e., subject-level variance 80%), presumably partly due to underlying genetic control (Dubois *et al.*, 2012), while psychiatric correlates show more change than stability over time (i.e., subject-level variance of ~23%). The latter also holds true for the lifestyle variables (i.e., subject-level variances ranging from 2% to 32%), presumably due to different ages of initiation of substance use with general absence in childhood and peaks in adolescence and young adulthood. With respect to epigenetic aging and cumulative childhood trauma, approximately half of the total variance can be attributed to differences between subjects, while the other half can be explained by changes between waves. In other words, epigenetic aging and exposure to childhood trauma are relatively stable but also show dynamic potential. Taken together, these results show that all study variables show both stability and change over time.

**Lagged changes in epigenetic aging**

Higher depression symptoms (b=1.67 months, P=0.003) and functional impairments (b=3.18 months, P=0.012) were associated with higher rates of epigenetic aging (**Supplementary** **Figure S5**). However, after multiple testing correction only depressive symptoms (P_FDR_=0.024) remained significant. **Supplementary Table S3** provides an overview of the epigenetic aging percentile distribution per health risk.

**Supplementary Figure S5. Lagged effects of health risks on epigenetic aging.** The *x*-axis shows percentile distributions of the health risk and the *y*-axis shows the change in epigenetic aging over time between two assessments in months. For example, persons with a depressive symptom score in the 99^th^ percentile showed 10 months of epigenetic aging on average. Models were residualized for Δ chronological age, adult age, sex, race/ethnicity, Δ body mass index, adult body mass index, Δ smoking, adult smoking, Δ cannabis, adult cannabis, Δ alcohol, adult alcohol, Δ estimated cell-type proportions, adult estimated cell-type proportions, and lab technical covariates. P-values were uncorrected for multiple comparisons.

**Supplementary Table S3.** Epigenetic aging percentile distribution per health risk.

|  |  | **Change in epigenetic aging (months)** | |
| --- | --- | --- | --- |
| **Distribution of health risk** | Percentile | *Depressive symptoms* | *Impairments* |
|  | 0.00% | -8.50 | -6.42 |
|  | 1.00% | -6.49 | -5.30 |
|  | 10.00% | -4.38 | -3.05 |
|  | 20.00% | -3.28 | -2.48 |
|  | 25.00% | -2.85 | -2.17 |
|  | 30.00% | -2.34 | -2.01 |
|  | 40.00% | -1.52 | -1.48 |
|  | 50.00% | -0.76 | -0.95 |
|  | 60.00% | 0.26 | -0.39 |
|  | 70.00% | 1.36 | 0.81 |
|  | 75.00% | 1.84 | 1.46 |
|  | 80.00% | 3.20 | 2.69 |
|  | 90.00% | 6.12 | 5.15 |
|  | 95.00% | 7.97 | 7.01 |
|  | 99.00% | 9.86 | 8.99 |
|  | 100.00% | 11.94 | 9.85 |

*Change in epigenetic aging is indicated in months and represents the change between childhood/adolescence (T2) and adulthood (T3).*

**Lagged changes in health risks**

To examine whether epigenetic aging in childhood/adolescence predicted changes in health risks, we also fitted regression models with epigenetic aging at childhood/adolescence as predictor and change in health risk (e.g., BMI in adulthood minus BMI at the latest available childhood/adolescent observation) as an outcome, while correcting for covariates at childhood/adolescence. There were no significant findings from the analyses examining whether epigenetic aging in childhood/adolescence predicted changes in health risks in adulthood (**Supplementary Table S4**)

**Supplementary Table S4.** Lagged effects of epigenetic aging on changes in health risk factors.

| **Health risk factor** | **b (in months)** | **95% CI** | **SE** | **t value** | **P** | **P_FDR_** |
| --- | --- | --- | --- | --- | --- | --- |
| BMI | -0.35 | -0.61 - -0.08 | 0.14 | -2.53 | 0.01 | 0.11 |
| Depressive symptoms | 0.01 | -0.06 - 0.08 | 0.04 | 0.37 | 0.71 | 0.99 |
| Anxiety symptoms | -0.08 | -0.19 - -0.02 | 0.05 | -1.52 | 0.13 | 0.58 |
| Childhood Trauma | 0.01 | -0.03 - 0.04 | 0.02 | 0.38 | 0.71 | 0.99 |
| Impairments | 0.01 | -0.17 - 0.19 | 0.09 | 0.11 | 0.92 | 0.99 |
| Poverty | -0.01 | -0.03 - 0.03 | 0.02 | -0.07 | 0.94 | 0.99 |
| Smoking | -0.01 | -0.03 - 0.02 | 0.01 | -0.12 | 0.90 | 0.99 |
| Cannabis | 0.00 | -0.01 - 0.01 | 0.01 | -0.02 | 0.99 | 0.99 |
| Alcohol | -0.01 | -0.02 - 0.00 | 0.01 | -1.22 | 0.22 | 0.67 |

Abbreviations: b, unstandardized beta; SE, standard error of the estimate; BMI, Body Mass Index. All models were corrected for chronological age, adult age, adult health risk values, sex, race/ethnicity, Δ estimated cell counts, adult estimated cell counts, and lab technical covariates. P_FDR_ indicates false discovery rate adjusted P-values.

**Overlap of our DNAm age predictor and other epigenetic clocks**

Epigenetic clocks, based on Illumina methylation platform data, have emerged as powerful tools for estimating DNAm age. However, the landscape of epigenetic research extends beyond these well-established clocks, incorporating diverse methylation data sources such as MBD-seq. We explored the intersections between our predictor and two widely recognized epigenetic clocks: the Horvath epigenetic clock (Horvath, 2013) and the Hannum epigenetic clock (Hannum et al., 2013). To assess the commonalities between our MBD-seq based predictor and the other epigenetic clocks we compared the methylation sites covered by our predictor with those identified by the Horvath and Hannum clocks. There was a degree of overlap between the CpG sites identified by our predictor and those featured in the Horvath (26/353) and Hannum predictors (8/77) and the overlapping DNA methylation sites and their associated weights can be found in **Supplementary Tables S5**. This convergence suggests at least a partly shared biological overlap. While it was our goal to obtain unbiased DNAm age predictions for our current sample and not to develop a new epigenetic clock, the 25K CpG sites and associated weights derived from our elastic net predictor are presented in **Supplementary Table S6**.

**REFERENCES**

Aberg, K. A., Chan, R. F., Shabalin, A. A., Zhao, M., Turecki, G., Staunstrup, N. H., … van den Oord, E. J. (2017). A MBD-seq protocol for large-scale methylome-wide studies with (very) low amounts of DNA. *Epigenetics: Official Journal of the DNA Methylation Society*, *12*(9), 743–750.

Aberg, K. A., Chan, R. F., & van den Oord, E. J. C. G. (2020). MBD-seq - realities of a misunderstood method for high-quality methylome-wide association studies. *Epigenetics: Official Journal of the DNA Methylation Society*, *15*(4), 431–438.

Aberg, K. A., Mcclay, J. L., Nerella, S., Xie, L. Y., Clark, S. L., Hudson, A. D., … van den Oord, E. J. C. G. (2012). MBD-seq as a cost-effective approach for methylome-wide assocciation studies: demonstration in 1500 case-control samples. *Epigenomics*, *4*(6), 605–621.

Angold, A., Prendergast, M., Cox, A., Harrington, R., Simonoff, E., & Rutter, M. (1995). The Child and Adolescent Psychiatric Assessment (CAPA). *Psychological Medicine*, *25*(4), 739–753.

Angold, Adrian, & Jane Costello, E. (2000). The Child and Adolescent Psychiatric Assessment (CAPA). *Journal of the American Academy of Child & Adolescent Psychiatry*, Vol. 39, pp. 39–48. doi:10.1097/00004583-200001000-00015

Canino, G., Costello, E. J., & Angold, A. (1999). Assessing Functional Impairment and Social Adaptation for Child Mental Health Services Research: A Review of Measures. *Mental Health Services Research*, *1*(2), 93–108.

Cecil, C. A. M., Zhang, Y., & Nolte, T. (2020). Childhood maltreatment and DNA methylation: A systematic review. *Neuroscience and Biobehavioral Reviews*, *112*, 392–409.

Chan, R. F., Shabalin, A. A., Xie, L. Y., Adkins, D. E., Zhao, M., Turecki, G., … van den Oord, E. J. C. G. (2017). Enrichment methods provide a feasible approach to comprehensive and adequately powered investigations of the brain methylome. *Nucleic Acids Research*, *45*(11), e97.

Clark, S. L., Hattab, M. W., Chan, R. F., Shabalin, A. A., Han, L. K. M., Zhao, M., … van den Oord, E. J. C. G. (2019). A methylation study of long-term depression risk. *Molecular Psychiatry*, *25*(6), 1334–1343.

Costello, E. J., Eaves, L., Sullivan, P., Kennedy, M., Conway, K., Adkins, D. E., … van den Oord, E. (2013). Genes, environments, and developmental research: methods for a multi-site study of early substance abuse. *Twin Research and Human Genetics: The Official Journal of the International Society for Twin Studies*, *16*(2), 505–515.

Dorn, L. D., & Biro, F. M. (2011). Puberty and Its Measurement: A Decade in Review. *Journal of Research on Adolescence: The Official Journal of the Society for Research on Adolescence*, *21*(1), 180–195.

Dubois, L., Ohm Kyvik, K., Girard, M., Tatone-Tokuda, F., Pérusse, D., Hjelmborg, J., … Martin, N. G. (2012). Genetic and environmental contributions to weight, height, and BMI from birth to 19 years of age: an international study of over 12,000 twin pairs. *PloS One*, *7*(2), e30153.

Han, L. K. M., Aghajani, M., Clark, S. L., Chan, R. F., Hattab, M. W., Shabalin, A. A., … Penninx, B. W. J. H. (2018). Epigenetic Aging in Major Depressive Disorder. *The American Journal of Psychiatry*, *175*(8), 774–782.

Hannum, G., Guinney, J., Zhao, L., Zhang, L., Hughes, G., Sadda, S., … Zhang, K. (2013). Genome-wide Methylation Profiles Reveal Quantitative Views of Human Aging Rates. *Molecular Cell*, *49*(2), 359–367.

Hattab, M. W., Shabalin, A. A., Clark, S. L., Zhao, M., Kumar, G., Chan, R. F., … van den Oord, E. J. C. G. (2017). Correcting for cell-type effects in DNA methylation studies: reference-based method outperforms latent variable approaches in empirical studies. *Genome Biology*, *18*(24). doi:10.1186/s13059-017-1149-7

Horvath, S. (2013). DNA methylation age of human tissues and cell types. *Genome Biology*, *14*(10), R115.

Houseman, E., Accomando, W. P., Koestler, D. C., Christensen, B. C., Marsit, C. J., Nelson, H. H., … Kelsey, K. T. (2012). DNA methylation arrays as surrogate measures of cell mixture distribution. *BMC Bioinformatics*, *13*(1), 86.

Koestler, D. C., Christensen, B., Karagas, M. R., Marsit, C. J., Langevin, S. M., Kelsey, K. T., … Houseman, E. A. (2013). Blood-based profiles of DNA methylation predict the underlying distribution of cell types: a validation analysis. *Epigenetics: Official Journal of the DNA Methylation Society*, *8*(8), 816–826.

Langmead, B., & Salzberg, S. L. (2012). Fast gapped-read alignment with Bowtie 2. Nat. 940. *Methods* , *9*(357–359), 541.

McKenna, A., Hanna, M., Banks, E., Sivachenko, A., Cibulskis, K., Kernytsky, A., … DePristo, M. A. (2010). The Genome Analysis Toolkit: a MapReduce framework for analyzing next-generation DNA sequencing data. *Genome Research*, *20*(9), 1297–1303.

Shabalin, A., Clark, S., Hattab, M., Aberg, K., & van den Oord, E. (2017). *ramwas: Fast Methylome-Wide Association Study Pipeline for Enrichment Platforms. R package version 1.0.0, https://bioconductor.org/packages/ramwas/*.

Tanner, J. M. (1962). The evaluation of growth and maturity in children. *Protein Metabolism*, pp. 361–382. doi:10.1007/978-3-642-53147-7_23

van den Oord, E. J. C. G., Bukszar, J., Rudolf, G., Nerella, S., McClay, J. L., Xie, L. Y., & Aberg, K. A. (2013). Estimation of CpG coverage in whole methylome next-generation sequencing studies. *BMC Bioinformatics*, *14*, 50.
